# Biased and Biasing: The Hidden Bias Cascade and Bias Snowball Effects

**DOI:** 10.3390/bs15040490

**Published:** 2025-04-08

**Authors:** Itiel E. Dror

**Affiliations:** Cognitive Consultants International (CCI-HQ), London, UK; Itiel@cci-hq.com

**Keywords:** cognitive bias, bias cascade, bias snowball, bias fallacies, sources of bias, Linear Sequential Unmasking (LSU-E), minimizing bias, implicit bias, forensic science, Swiss cheese model

## Abstract

Cognitive bias is widespread, hidden, and difficult to deal with. It impacts each and every aspect of the justice and legal systems, from the initial engagement of police officers attending the crime scene, through the forensic examination, and all the way to the final outcome of the jurors’ verdict and the judges’ sentencing. It impacts not only the subjective elements in the justice and legal systems but also the more objective scientific elements, such as forensic fingerprinting and DNA. The impact of bias on each of these elements has mainly been researched and considered in silo, neglecting the biasing interactions and how bias cascades and snowballs throughout the justice and legal systems. These should happen rarely, as the Swiss cheese model shows that such errors in the final outcome rarely occur because they require that the shortcomings in each element be coordinated and aligned with the other elements. However, in the justice and legal systems, the different elements are not independent; they are coordinated and mutually support and bias each other, creating and enabling hidden *bias cascade* and *bias snowball* effects. Hence, minimizing bias requires not only taking measures to reduce bias in each of the elements but also a wider perspective that addresses bias cascade and bias snowball effects.

## 1. The Hidden Bias Cascade and Bias Snowball Effects

It is well established that the justice and legal systems are plagued with bias. Given the human role and subjectivity in policing, eyewitnesses’ accounts, jurors’ decision making, and judges’ sentencing, it is not surprising that bias impacts the different elements of the justice and legal systems (Berthet, 2022; Simon, 2012; Vidmar, 2011; Voigt et al., 2017). What is less known and researched is how these biases impact even the ‘objective’ and ‘scientific’ elements of the justice and legal systems.

Furthermore, the biases within these elements have been examined mainly in silo, without their interactions that create bias cascade and bias snowball effects. In this paper, I will first elaborate and explain cognitive biases, as they are a great concern due to their widespread implicit impact. Then, I will show their sources and effects throughout the various elements of the justice and legal systems. Moving from bias in specific elements, I will demonstrate how the biases are magnified by bias cascade and bias snowball effects. The paper will then end with practical ways to minimize such biases.

### Explicit and Intentional Bias

Bias has many forms and many manifestations. On the face of it, explicit intentional bias is the worst (Fiske, 2004; Daumeyer et al., 2019). When explicit intentional biases occur in the justice and legal systems it is especially alarming as people can be wrongfully convicted, and conversely, guilty people can go free. Indeed, it has been shown that there are cases of explicit and intentional bias in a variety of elements in the justice and legal systems (e.g., Burke et al., 2024).

Feeding some of these unethical biased behaviors are people in the justice and legal systems who believe so much in what they are trying to achieve that they feel justified to take such actions (e.g., ‘the end justifies the means’, ‘the greater good’, ‘morally wrong actions are sometimes necessary to achieve morally right outcomes’, etc.). This is known as ‘The Dirty Harry Problem’ (Klockars, 1980) and ‘Noble cause corruption’ (Delattre, 2011; Grometstein, 2007), and it can impact policing and legal proceedings, as well as forensic work (Wienroth & McCartney, 2023).

Perhaps most alarming is when ‘objective’ and ‘scientific’ elements in the justice and legal systems, which are supposedly impartial and not biased (and present themselves as such), also exhibit intentional biases. For example, signal detection analysis shows that forensic science experts in firearms evidence *deliberately* do not report elimination conclusions (Smith & Wells, 2023). This explicit intentional bias of not reporting exculpatory evidence has serious implications for the justice and legal systems because it deprives innocent suspects of the evidence they need to prove their innocence (Smith & Wells, 2023). If forensic scientists who are supposedly impartial and objective show such intentional and explicit bias, then it is no wonder or surprise that the more subjective and less scientific elements of the justice and legal systems have such biases too.

While such explicit intentional bias is a major concern, this article rather focuses on cognitive bias, which I view as an even greater concern because of the following:

1. It is widespread. Although explicit intentional bias exists, it is not as common and widespread as cognitive bias. Whereas the former is exhibited only by some ‘bad apples’ who are deliberately and intentionally biased, the latter cognitive bias is a ubiquitous phenomenon that impacts everyone due to the top-down nature of human cognition and other aspects of cognitive architecture (Nickerson, 1998).
2. It is harder to detect. Explicit intentional bias is much easier to detect relative to implicit hidden cognitive bias. The very nature of implicit bias makes it less apparent, and thus this bias is harder to detect than explicit intentional bias. Furthermore, it is not only implicit but it is also often not even within the conscious awareness of the person who is exhibiting the bias. Indeed, indirect measures are required where implicit cognitive bias is concerned (Greenwald & Banaji, 1995).
3. Minimizing and countering cognitive bias is not easy or straightforward. The ‘bad apples’ who exhibit explicit intentional bias are relatively easy to deal with. However, cognitive bias poses a bigger challenge (e.g., Hetey & Eberhardt, 2018).

Thus, without diminishing the issue of explicit intentional bias, this paper focuses on the more challenging, more widespread, and harder to detect and deal with cognitive bias.

## 2. What Is Cognitive Bias?

Cognitive bias is an outcome, a by-product, of people’s cognitive architecture and of how the brain processes information. Within the justice and legal systems, cognitive bias has been defined as “the class of effects through which an individual’s pre-existing beliefs, expectations, motives, and situational context influence the collection, perception, and interpretation of evidence during the course of a criminal case”^1^ (Kassin et al., 2013, p. 45).

One of the fundamentals of human cognition is the use and reliance on top-down information (what we expect, our past experiences, our ideology and motivations, etc.; this is in contrast to bottom-up information, which is the actual input data we receive). As information arrives in the brain (the bottom-up information), the top-down processes guide if and how to process that incoming information. The top-down brain–cognitive processes impact not only our judgments but also what we ‘see’ (Balcetis & Dunning, 2006), e.g., selective attention (Beilock et al., 2002; Deutsch & Deutsch, 1963; Myles-Worsley et al., 1988; Treisman, 1961; Wood, 1999).

It is important to distinguish between random ‘noise’ and systematic biases (Kahneman et al., 2021), created by top-down influences, such as motivation, past experiences, expectations, and a whole range of pre-existing information (e.g., Ask & Granhag, 2005; Lange et al., 2018; Nickerson, 1998). These top-down processes, by their very nature, can cause bias because the actual data/evidence is processed not only based on the merit and characteristic of the ‘input’/data itself but by a whole range of other factors that mediate if and how the input data are processed.

It is important to note that these top-down processes are essential and indispensable for human cognition. They make information processing efficient and are part of the basic architecture of the brain to meet the computational demands of human cognition relative to the capacity of the brain. It is also important to emphasize that many of these top-down influences occur automatically and without awareness (Kunda, 1990; Nickerson, 1998; Nisbett & Wilson, 1977; Wilson & Brekke, 1994).

The top-down nature of human cognition and the architecture of the human brain make cognitive bias widespread. Nevertheless, there is often denial and pushback by the justice and legal systems when the issue of bias is raised (e.g., Brook et al., 2021; Peat, 2021). This is partially due to the fact that cognitive bias impacts hard-working and dedicated people who are not aware of their biases (the *bias blind spot* means that people are relatively blind to their own biases (Pronin et al., 2004; see also Neal & Brodsky, 2016).

Because these cognitive biases are different and distinct from explicit intentional biases, they should entail a different approach. We need to understand their specific sources within the justice and legal systems, which will then enable us to develop ways to minimize their impact. However, we cannot move forward before we dismiss and do away with widely held incorrect conceptualizations and fallacies about cognitive bias (I. E. Dror, 2020).

### 2.1. Common Misconceptions and Fallacies About Cognitive Bias

When it comes to cognitive bias, there are a number of misconceptions and fallacies (I. E. Dror, 2020). A common fallacy is that cognitive bias is an ethical issue of personal integrity or incompetence (Moore et al., 2005, 2010). It is a fallacy to understand cognitive bias as a matter of personal character or/and incompetence. This misconception is apparent when cognitive bias is incorrectly attributed to a person’s immoral character. Cognitive bias impacts honest, hard-working, competent, and dedicated people.

Another common fallacy is that expertise and experience reduce and protect (or even immunize) from the effects of cognitive bias. Often, people within the justice and legal systems claim that they are not biased because of their vast and extensive experience. On the contrary, experience does not reduce bias, and it can actually increase or create bias because it enhances the prevalence, power, and impact of the top-down processes mentioned earlier (e.g., more selective attention, more a priori expectations, chunking, etc.—see I. E. Dror, 2011). Furthermore, with expertise, base-rate bias is likely to increase (see below). Indeed, research has shown that experts in the justice and legal systems are just as susceptible to bias as novices (e.g., Eeden et al., 2019).

With advances in AI and technology, a growing fallacy is that the mere use of technology and AI, per se, protects from bias. It is important to clearly and explicitly make the point that this is a fallacy. AI and technology do not necessarily give protection from bias (Benjamin, 2019a, 2019b; I. E. Dror, 2020). Indeed, biases have already been observed in a variety of technologies and AI, e.g., in face recognition (Buolamwini & Gebru, 2018; Haber, 2021). The point is that it is a fallacy that technology necessarily protects from bias because the biases, for example, can be within the software (Haber, 2021; Noble, 2018).

Finally, a common fallacy is the illusion of control. The belief that people can eliminate their biases by mere willpower. Such efforts are noble and well-intended but they are ineffective from a cognitive perspective (Lynch et al., 2022). In fact, intentional efforts to minimize bias can actually increase the impact of the bias due to ironic and rebound processing (Wegner, 1994). To minimize cognitive bias, specific steps and actions must be taken (which are elaborated below)—awareness of the bias, per se, does not stop it.

### 2.2. Cognitive Biases in the Justice and Legal Systems and Their Sources

Bias plagues the justice and legal systems from the initial police officers arriving at a crime scene, through the lawyers and the evidence presented in court, all the way to the jurors’ decision and the judges’ sentencing, and even later at parole boards (Berthet, 2022; Berryessa et al., 2023; Curley et al., 2022a, 2022b; Danziger et al., 2011; Hetey & Eberhardt, 2018). Below, I briefly provide instances of how such biases have an impact throughout the justice and legal systems, and present them within a conceptual taxonomy in a way that helps understand them and develop ways to minimize their impact.

Thus, rather than just listing biases (as often occurs in the literature), I will present them according to their source and their underpinning. This framework explicates and characterizes the different sources of biases and has been originally developed and applied within the specific domain of forensic evidence (I. E. Dror, 2020). Below, it is expanded, showing its wider impact and extensive applicability across the legal and justice systems.

**Figure 1:**
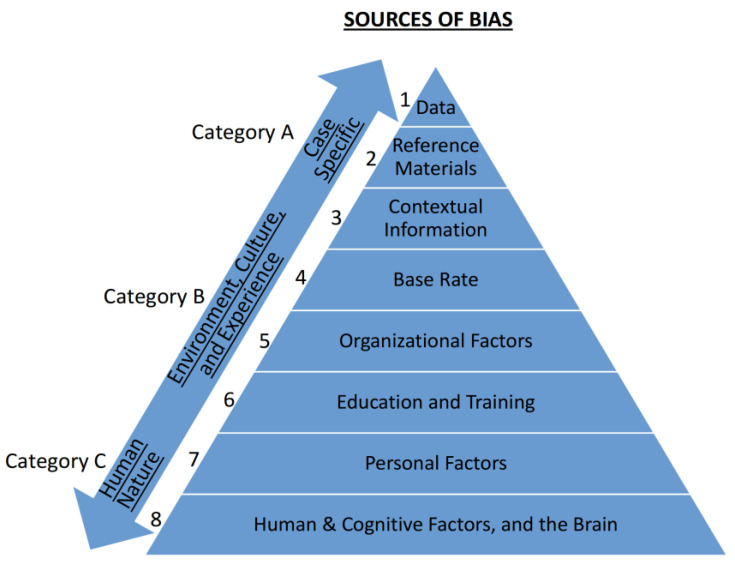
The eight sources that may bias observations, judgements, and decisions throughout the justice and legal systems. They are organized in a taxonomy within three categories: starting at the top with biases emerging from the specific *case* (Category A); moving down to biases emerging from the specific *person* handling the case (Category B); and at the very bottom (Category C), biases emerging from *human nature* that impact all of us, regardless of the specific case or the specific person who is handling it (I. E. Dror, 2020).

There are eight sources of bias, which fall within three categories (see Figure 1). The first category (A) relates to the case at hand. Something about this *specific case* triggers the bias. The second category (B) relates to the person handling the case rather than the case itself—it is the *specific person* handling the case that gives rise to the bias. Lastly, in category (C), the source is not the specific case or the specific person doing the work but human nature, which affects all of us regardless of the case and the person doing the work.

The first source of bias is the actual evidence, the Data (Level 1 in Category A). It can create empathy and sympathy (for example, with the victim or/and their family), which motivates them to solve the case and have cognitive closure (Ask & Granhag, 2005). Furthermore, disliking or feeling sympathy for the defendant, and disgust or anger toward the offense, may also create bias (Neal & Brodsky, 2016). It is only natural that horrific crimes and cases of child abuse provoke emotions and may bias observations and judgments (Almada et al., 2021).

The second source of bias within the specific case information is the Reference Materials (Level 2). Reference materials can be the person that needs to be identified (e.g., what they look like, their fingerprint or DNA profile). Such reference materials are sometimes needed, and when not used properly, they create bias. The bias emerges when the reference materials, rather than the actual evidence, guide and drive the cognitive process. This creates backward reasoning and/or circular reasoning, i.e., a target-/suspect-driven bias.

For example, in instances of identification by non-eyewitnesses (people who did not actually observe the crime but nonetheless testify about who they think is depicted in a video footage of the event based on their prior familiarity with that person (Pezdek & Lerer, 2023), it is important not to let the reference material bias this identification. That is, the witness should view the video footage and be asked if they can identify ‘anyone’, rather than asking them if they can identify ‘person X’. By asking them if they can identify ‘person X’, the reference material (in this case the suspect X) is primed and the cognitive process is driven by X, i.e., look for X in the video, rather than letting the actual evidence (the video footage) drive the cognitive process, without expectation and priming of who might be present. Similarly, jurors should hear audio recordings initially without the accompanying transcripts because the transcripts act as reference materials, whereas the evidence itself is the audio recording in question (more details on ways to minimize these biases are detailed below).

Such biases can even emerge in the most scientific aspects of justice and legal systems, such as fingerprinting and DNA (I. E. Dror, 2018). In fingerprinting, one must first examine, characterize, and document the latent print from the crime scene and only thereafter be exposed to the fingerprint of the suspect—not the other way around (knowing the fingerprint of the suspect and then trying to see if it is in the evidence). Similarly, knowing the DNA profile of the suspect can cause bias in developing the mixture of DNA profile from the crime scene (the evidence), so it fits the suspect’s DNA profile (Jeanguenat et al., 2017).

These and other biases occur to a large extent because of what I call the *forensic degrees of freedom*: forensic examiners (even in the more scientific forensic domains) have latitude for discretion and judgment, i.e., degrees of freedom in their decision making. As there are more degrees of freedom, bias has more opportunity to impact the decision making. Forensic degrees of freedom exist at different levels depending on the specific forensic domain.

This second source of biases (Level 2) arises in many aspects across the justice and legal systems, from police investigations (Baldwin, 1993; Wagenaar et al., 1993) to evidence about shaken baby syndrome (Lynøe et al., 2017). Thus, such use of reference materials causes motivation and expectations that then can bias the observations and interpretation of the actual evidence (e.g., Ask & Granhag, 2005; Balcetis & Dunning, 2010; Kunda, 1990; Lange et al., 2018).

The third and last source of bias within Category A is Contextual Information (Level 3). Often people in the justice and legal systems know a lot of irrelevant background contextual information that can bias them. For example, fingerprint experts whose job is to compare two fingerprints (one from the crime scene and one from the suspect) and to decide if they are ‘similar enough’ to conclude that they originate from the same source are exposed over 40% of the time to information about the suspect’s criminal history (Gardner et al., 2019). The problem is that such contextual irrelevant biasing information can dramatically affect decisions and increase the risk of convicting an innocent person (Thompson, 2023).

In the justice and legal systems, we can see such biases in police interviews and interrogations. For example, in child sexual abuse, where the interview statements of alleged victims often become the primary source of evidence, the prior beliefs of the interviewer can cause them to ask suggestive questions and pursue the interview in directions that fit and correspond to their prior beliefs (Huang & Bull, 2021).

The power of such contextual irrelevant information has even been demonstrated in the most used and highly regarded evidence in the justice and legal systems, e.g., DNA and fingerprinting (I. E. Dror & Hampikian, 2011; I. E. Dror et al., 2006). For example, testing expert fingerprint examiners showed that they can reach different and conflicting conclusions on the same evidence when presented within biasing irrelevant context. (I. E. Dror et al., 2006; for a review, see Kukucka & Dror, 2023). If cognitive bias impacts scientific evidence (see the *forensic degrees of freedom*, above), then it surely impacts (as much, if not more) less scientific elements in the justice and legal systems, be it judges, jurors, police, attorneys, and witnesses.

Contextual information can have such a strong and biasing effect to a level that it may not only determine the decisions (when identical evidence is presented) but it can even override the evidence-based decision, so the decision is modified to fit the contextual information (I. E. Dror et al., 2022)—this can occur especially in the presence of bias snowball, which is explained below. This bias, of course, has huge implications across the justice and legal systems; for example, forensic pathologists may be biased to avoid rendering a homicide manner of death when a person dies while in police custody; concluding the death as ‘natural’ or ‘undetermined’ often avoids a full investigation of the circumstances (and perhaps responsibility and accountability) for their death (Shapiro & Keel, 2023).

All the above three sources of bias (Levels 1–3) relate to the *specific case* at hand (Category A). The next set of sources (Category B) has nothing to do with the specific case; rather, they relate to and emerge from the *specific person* doing the work. The first source in this category is Base Rate (Level 4). People who work in the justice and legal systems have past experiences. These may cause a bias due to the *prevalence effect* (the phenomena whereby people are more likely to miss and not see things that have a lower prevalence base rate, i.e., things that are rare and infrequent, Biggs et al. (2018) and Godwin et al. (2015); and specifically in the justice and legal systems, Growns and Kukucka (2021)).

The base rate may also further create top-down expectations that create a bias (Lange et al., 2018). For example, a pathologist may have experienced that children who died while in the care of the mother’s boyfriend have been more frequently determined to have died as a result of homicide rather than an accident (relative to children who died while in the care of a family member). Given identical medical information, these base rate past experiences may bias forensic experts to determine homicide or accident based solely on who was the caregiver (I. E. Dror et al., 2021). Indeed, death certificate data show that black children, relative to white children, are more likely to have homicide listed as their manner of death, which can create a base rate bias when determining future manner of death of children (I. E. Dror et al., 2021).

Such biases are prevalent across the justice and legal systems. For example, police officers may perceive a higher base rate for finding drugs when they stop and search black male teenagers (Vomfell & Stewart, 2012). The bias then causes them to stop and search black male teenagers more, which then creates more ‘hits’ and a perceived higher base rate with black male teenagers, which only feeds and strengthens their bias—a self-perpetuating bias and a self-fulfilling prophecy. Of course, this base rate bias is not limited to police officers’ bias in stop and search but impacts many elements in the justice and legal systems.

Organizational Factors are the next source of bias (Level 5). There are many biases that emerge from organizational pressures and cultures. For example, implicit prosecutorial biases or working within and for a specific side can create bias (Winburn & Clemmons, 2021). Organizational factors are especially acute in an adversarial legal system, as adversarial allegiance and mindset can cause judgments and conclusions (about the same identical evidence) to depend on which side retained them. Murrie et al. (2013) describe it as the tendency to reach conclusions that support the party who hired them (see also Melnikoff & Strohminger, 2020; Simon et al., 2020).

Training and Education (Level 6) have a long-term impact. For example, it has been shown that judges have biases that may have been shaped by their training and education as prosecutors (Berryessa et al., 2023; see also Vidmar, 2011). Another example in the wider context of the justice and legal systems is police officers who are often trained to presume a suspect is guilty. Such a suspicion against suspects creates bias and generates evidence (such as false confessions) that can lead to wrongful convictions (Baldwin, 1993; Inbau et al., 2001; Kassin et al., 2003).

Personal Factors (Level 7) pertain to the personal experiences, beliefs, ideology, political views, and personality of the person making the decision. When it comes to jurors, for example, there are plenty of biases that impact their judgments and decisions, from the race of the defendant to the victim’s attractiveness (Maeder et al., 2015). Such attitudes and biases are so powerful that they enable to predict jurors’ verdicts (Lecci & Myers, 2008, 2009). These biases may undermine the justice and legal systems’ goal of providing unbiased justice (Curley et al., 2022b).

These biases also impact police officers, prosecutors, and judges who may be dealing with a type of case that they themselves have been a victim of (or know someone who has). Consider a child sexual abuse case where the police detective, forensic examiner, prosecutor, or judge dealing with this case have themselves been a victim of such a crime (Goldenson & Gutheil, 2023). This can cause biases within the wider justice and legal systems. However, on the flip side, their personal experience may also give them an epistemic advantage with unique understanding and insights (L. Dror, 2022). Some in the justice and legal systems may even be on a ‘crusade’, justifying their biased actions because the ‘end justifies the means’ (see ‘Noble cause corruption’, above).

Also, given that the justice and legal systems deal with social issues, these relate and are inherently connected to values and ideologies (Frank, 1930; Winburn & Clemmons, 2021). For example, some may have a more conservative viewpoint, believing in ‘tough on crime’ and emphasizing punitive actions, whereas others may have a more liberal viewpoint, believing in rehabilitation and emphasizing human rights. Some may be more compassionate and sympathetic to the victims and their rights, whereas others are more concerned with the rights of the accused. Such personal factors—and there are many more of them beyond beliefs, ideologies, and values– can bias and impact the work of various elements across the justice and legal systems.

Even “judges’ decisions vary according to their personal backgrounds and, more importantly, according to their ideology” (Harris & Sen, 2019, p. 241). Since the justice and legal systems deal with and relate to social issues, personal values, ideologies, and motivations—maybe even why some people go into this profession in the first place– will all create biases and impact objectivity (Guthrie et al., 2001; Vidmar, 2011; Winburn & Clemmons, 2021).

Finally, Category C (Level 8), has nothing to do with the specific case or the specific person doing the work, but relates to human nature, the architecture of human cognition, as well as human emotional needs, as briefly discussed earlier this can relate to top-down cognitive processes, selective attention, the need for closure, confirmation bias, etc. (e.g., Danziger et al., 2011; Nickerson, 1998).

We have seen how these sources of bias (which are not mutually exclusive, Neuilly, 2022) impact various elements across the justice and legal systems. However, their impact goes beyond individual elements of the justice and legal systems, as bias cascade and bias snowball are created by their hidden interactions, which are presented below.

## 3. Bias Cascade and Bias Snowball Effects

Biases in the justice and legal systems have been mainly researched and considered in silo. However, the combined biases, across different elements, possess hidden biases, as their interactions create bias that is more than their sum. The reason for this is that elements in the justice and legal systems (e.g., the police, the forensic examiners, and the prosecution), instead of working independently they work in concert, influencing and creating biases that mutually support one another.

Working independently enables checks and balances that create safeguards and protection. For example, the police theory about what happened at the crime scene and who is guilty of the crime may be refuted by scientific experts. Similarly, the attorneys at the District Attorney (DA) prosecution office examine the evidence provided by the police and forensic analyses in order to determine whether to go ahead and prosecute. These checks and balances make sure that if the evidence is biased (or other possible faults), it is caught and stopped from going any further.

**Figure 2:**
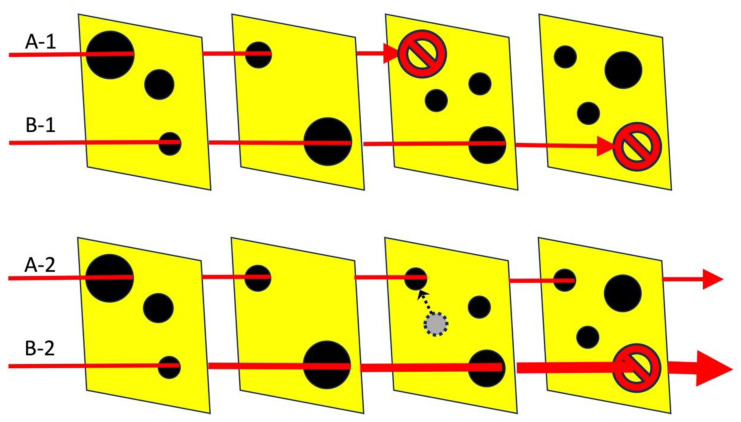
An adaptation of the Swiss cheese model (Reason, 1990). Top panel: although bias exists in the various elements within the justice and legal systems (the black holes in the Figure), it rarely impacts the final outcome because a bias has to be aligned through all the different layers. E.g., ‘A-1’ in the top panel has been able to go through two layers but becomes stuck and is stopped at the third layer; similarly, ‘B-1’ gets stuck and ends at the fourth layer. As the justice and legal systems have many elements before someone is convicted (many checks and balances even before it goes to court), it would rarely happen that a bias could penetrate and align through all the various elements. However, this all hinges on and assumes that the different elements are relatively independent from each other. As illustrated in the bottom panel, the reality is that supposedly independent elements (that should constrain one another) actually work in concert, influencing and biasing each other, creating bias cascade and bias snowball effects (which are not mutually exclusive). ‘A-2’ is able to go through all the layers (in contrast to ‘A-1’) because the *bias cascade* has moved the hole in layer three to align with the holes in layers 1 and 2. ‘B-2’ illustrates *bias snowball* in which multiple biases support and feed each other, creating a bigger and bigger bias so powerful that it can penetrate and go through the last layer.

The Swiss cheese model (Reason, 1990), which has been widely and extensively used in many domains, illustrates that even when one element has a fault (see the ‘black holes’ in Figure 2), be it bias or another fault, it, by itself, will not result in an error in the final outcome. This is because the fault will be stopped by the checks and balances carried out by the other elements (see the ‘slices of cheese’ in the top panel of Figure 2). For an error to occur in the final outcome, the fault has to be aligned across all the elements—something that is unlikely and a relatively rare event.

The Swiss cheese model was developed and has been used in accident investigation and prevention, with widespread acceptance in aviation, healthcare, and across many other domains. In a nutshell, many systems have multiple checks and balances, and therefore, if one of them fails, it will nevertheless not lead to a negative outcome, as long as it is caught and not allowed to propagate and to end up in a resulting accident.

This has been illustrated as layers of Swiss cheese, each one with its own flaws that enable a failure (the holes in each layer, see Figure 2). Even with the holes in each layer, a final negative outcome is nevertheless unlikely because the next layer (of checks and balances) will not have aligning corresponding holes. Thus, an accident and other negative outcomes can be minimized by structures that have multiple layers of checks and balances.

The justice and legal systems are an excellent application of the Swiss cheese model because they have multiple layers of checks and balances. Within the justice and legal systems, the ‘holes’ in the layers of cheese do not only represent flaws, defects, or errors (as in the original model) but they also represent intentional or built-in biases that exist and are inherent to the justice and legal systems (e.g., its adversarial nature). With such ‘holes’ in each layer, i.e., even if each layer is not intended to be objective, then those different biases are supposed to counter each other across layers.

In the application to the justice and legal systems, for an innocent person to be convicted, errors, biases, and other shortcomings have to occur and be aligned across many elements of the justice and legal systems (be it the police investigation through the forensic and other evidence, as well as the during trial). A fault going through all this series of checks and balances without being detected and stopped is an unlikely event.

This type of system works well if the various elements are independent. For example, if the police are biased, the forensic examination (being performed independently without the impact of the police briefing and biasing them), will render conflicting results that will question the police theory. However, if the police and the forensic experts work as a team, interact, coordinate, and influence one another, the police bias may cascade and impact the forensic examination, creating matching and aligned biases. The initial idea of bias cascade and bias snowball was created within the specific domain of forensic science (I. E. Dror, 2018, 2020); here, they are developed and expanded conceptually, as well as applied and showing their impact across the entire justice and legal systems.

**Figure 3:**
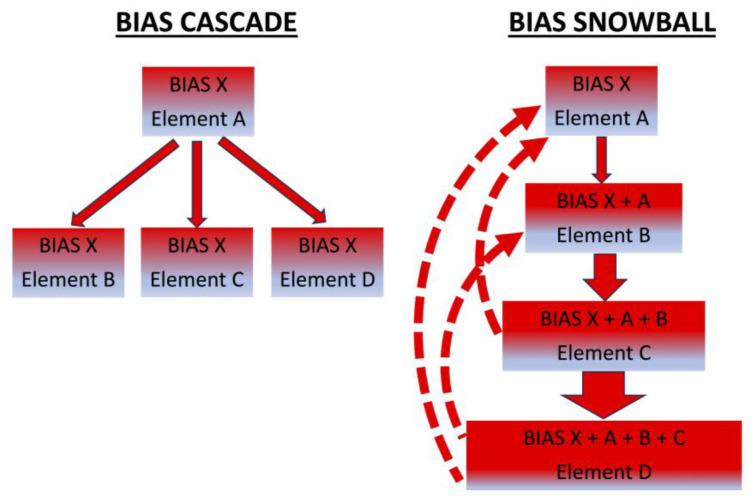
Bias Cascade (**left panel**) occurs when a bias is introduced and it not only contaminates and impacts that specific element (‘X’ biasing ‘A’ in the left panel of the Figure) but then that same bias (‘X’) cascades to also impact other elements (‘B’, ‘C’, and ‘D’, left panel). Bias Snowball (**right panel**) occurs when biases accumulate and feed one another. The impact increases as the bias gains greater and greater momentum with more and more biases added. In bias snowball, one bias creates additional new biases, which then support the creation of even more additional new biases, and so forth (including interactive loops adding more bias—the dashed arrows). As different elements of the justice and legal systems are being biased, they then bias others (on the right panel, once ‘A’ is contaminated by ‘X’, then ‘B’ is then contaminated by ‘X’ and by ‘A’; by the time it gets to ‘D’, biases ‘A, ‘B’, and ‘C’ have been created in addition to ‘X’). As this interactive contamination continues, the biases grow and grow, creating the Bias Snowball effect.

Bias cascade (see Figure 3, left panel) occurs when a bias that is introduced to an element in the justice and legal systems does not only impact that element. The bias cascades and thus also has an impact on other elements in the justice and legal systems. Consider, for example, that a Crime Scene Investigator (CSI) learns that the suspect is black or that they have a criminal record; this irrelevant contextual information creates a bias that impacts their work, e.g., the samples that they collect at the crime scene (Eeden et al., 2019; Lange et al., 2018). Bias cascade occurs when this biasing information does not only impact the CSI work but when the biasing information is cascaded to bias other elements in the justice and legal systems. For example, they share irrelevant information about the suspect’s past criminal record with the fingerprint examiner, witnesses, and others. Thus, this bias not only impacts the CSI but the bias cascades across the justice and legal systems.

Continuing with a CSI example, if they are briefed that a person died as a result of homicide (a biased brief, as the person actually died as a result of suicide), this bias may not only bias them to not collect critical evidence that suggests (or even proves) suicide (Eeden et al., 2019; Lange et al., 2018). This biased briefing can also be shared and thus cascaded to the forensic pathologist doing the autopsy. Just like the CSI was briefed before seeing the evidence themselves (so they can, at least initially, reach a non-biased conclusion based on the evidence—more details in the section that follows), so does the forensic pathologist who is most often briefed before conducting an autopsy. The biasing contextual brief received by the forensic pathologist originated and cascaded from the earlier bias initiated by the police briefing to the CSI. Such contextual biasing information can override the actual medical and autopsy findings (see, I. E. Dror et al., 2022, and the earlier discussion about the impact of Contextual Information, Level 3 in Figure 1).

Consider how bias cascade can impact a forensic anthropologist needing to determine in the laboratory the sex of skeletal remains based on the anthropological measurements of the skeleton evidence. Hartley et al. (2022) found that their decisions are influenced by irrelevant non-skeleton information cascading from those who recovered the skeleton from the crime scene, thus biasing the laboratory assessment of the skeletal remains (e.g., a photograph of the person, whether they were wearing male or female clothing, etc., i.e., evidence that is not about the anthropological measurements of the skeleton).

Bias cascade is present and prevalent throughout the justice and legal systems. In police interviews, whether of a victim of a sexual assault or child abuse, where the credibility of statements of alleged victims is critical and assessed by experts conducting the interviews, their assessments can be highly influenced by irrelevant prior biases cascading into how the interview is conducted and the conclusion reached (see earlier discussion). This bias cascade does not only impact how the interview is conducted and the assessment of the alleged victim’s statement but the same bias can also impact the interrogation of the suspect, which can lead to false confessions (Kassin, 2022).

Similarly, Bloodstain Pattern Analysis (BPA) experts can receive biasing contextual information cascading from other elements. This information is most often given *before* they even see the actual evidence (i.e., the bloodstain pattern), even though this potentially biasing information can (and should) all be minimized and sequenced correctly (details below). The bias cascade occurs when additional elements are biased by being exposed to a previous bias, which is cascaded and thus propagated to impact additional elements in the justice and legal systems.

Bias cascade also pertains to the verification of examination results. When the verification is not blind (i.e., the verifier knows what the original examiner’s results are, as well as all the contextual biasing information), then such so-called verifications are actually biased and not properly independent because the causes and biases that underpin any errors in the original results will just repeat themselves during the verification. Recently, a Circuit Court in the State of Oregon excluded evidence due to such bias (18 April 2024, Country of Multnomah, Case No: 22 CR57267) where the court ruled that: “there are reasons to question the value of the verifier’s conclusion because the verifier was aware of the prior examiner’s conclusions before performing their analysis” and therefore that “the State may not present testimony or other evidence that [name of the forensic examiner] conclusions were verified by another firearm’s examiner”.

The idea in bias cascade is that a specific bias is not limited to only impact one element in the legal and justice systems but that this specific bias cascades to impact other elements (see Figure 3, left panel, bias X does not only impact element A but bias X cascades to also impacting elements B, C, and D).

In bias snowball, the impact of the bias grows because those who have been biased now go to bias others as well; as more and more elements are impacted, they then also add new biases that impact other elements, creating a bias snowball. See Figure 3 (right panel), where once bias X impacts element A, then now element B is biased both by the original bias X cascading, as well as also by the added bias of element A, now thus exposing element B to bias X + A; the bias snowball continues to grow as element C will be biased by X + A + B, as well as circular loops of bias interactions between the different elements.

Bias snowball (see Figure 3, right panel) occurs when bias is not just cascading but additional biases are added (as different elements are being biased, they then add a bias that further impacts others), thus increasing the biases as it progresses through the justice and legal systems. Bias snowball is not like bias cascade, which is about one bias cascading to impact other elements, but bias snowball involves more and more new biases being added. As the biases accumulate and interact with one another, they add up and increase in power.

The multiple biases do not only add up but they continue to get stronger with multiple biases that generate greater momentum, which creates additional new biases. Hence, bias snowball has an active process in which an element that has been biased now actively adds its own bias to impact others. As more and more elements are being biased and then biased by others, the bias grows, hence the bias snowball.

The bias snowball is not as linear as the bias cascade where the bias merely cascades throughout the elements in the justice and legal systems. In the bias snowball, interactive circular loops often occur. Hence, bias snowball is not multiple bias cascades working independently alongside one another. For example, the District Attorney prosecution office does not receive the police and forensic reports to evaluate whether to prosecute and move to trial; rather, they go back and influence the previous elements. It is not about asking for legitimate clarifications but more about introducing pressure and bias to ‘re-do’ and ‘re-consider’ their work and conclusions or ‘just’ to ‘re-word’ parts of their reports, all aimed to make a stronger case against the suspect. This applies to defense attorneys too, who may try to influence and bias their experts and witnesses, so as to make their client seem more innocent.

However, with the prosecutors, there is the danger that additional biases, e.g., racial bias (Davis, 2007), will be introduced by the non-linear loops to create bias snowball throughout the justice and legal systems. In the bias snowball effect, the interactions of loops and circular influences cause the biases to mutually reinforce and amplify one another.

Consider a DNA examiner who is biased by knowing that an eyewitness identified the suspect as a contributor to the DNA mixture. This can bias their judgment and conclusion (as detailed earlier). The eyewitness identification can not only cascade to other elements of the justice and legal systems (e.g., making the fingerprint examiner also aware of the eyewitness identifications) but it can snowball if the fingerprint examiner is now not only aware of the cascading witness identification but also, in addition, to the DNA results as well. Thus, in the bias snowball effect, not only are different elements in the justice and legal systems biased by the same biasing information (the bias cascade) but in the snowball bias effect, the elements that have been biased add new biases that then go on to bias others (for a probabilistic formalization, see Cuellar et al., 2022).

Bias snowball occurs frequently in the justice and legal systems because elements that should be independent actually work together (e.g., Brook et al., 2021). Furthermore, the bias snowball creates unnecessary circular biasing loops in what is generally a linear process. The implication of the bias snowball effect is not only responsible for growing and increasing bias but also for misleading the court because it causes the double counting of evidence. In the example above, the eyewitness accounts will be double-counted (if not more) in court because the fact finder will not only get the eyewitness account explicitly and directly from their testimony, but they will also count the eyewitness account again, indirectly, via the conclusions and testimony of the DNA expert (which includes the effect of the eyewitness account); thus, the eyewitness account is double counted.

In terms of the Swiss cheese model, in bias cascade, the strength of the arrow does not change, i.e., the biasing information is the same, but the arrow is enabled to go through to impact and bias multiple layers/elements due to the ‘holes’ being coordinated so they are aligned to allow the arrow to go through (see A-2 in the bottom panel of Figure 2). In contrast, in bias snowball, the strength of the arrow increases as it goes through the various layers/elements; with the increase in strength, it is able to go through even smaller and smaller ‘holes’ to the extent of even penetrating and going through layers that do not have a corresponding aligning ‘hole’ (see B-2 in the bottom panel of Figure 2).

An example of that in the justice and legal systems is when the bias is so powerful that it will override the evidence-based decision, so an existing evidenced-based decision (e.g., did the person die as a result of homicide or suicide) is modified to fit the bias (I. E. Dror et al., 2022). There are many other examples of this throughout the justice and legal systems; some examples are how false confessions will override evidence, rewrite witnesses’ memories, and corrupt eyewitness identifications, as well as how they can cause forensic scientists to erroneously identify a suspect (Hasel & Kassin, 2009; Kassin et al., 2013; see also the cases of the Central Park Five or Brendan Dassey).

These biases are not mutually exclusive and they are often hidden, as there is little transparency about the interactions, influences, and coordination between different elements of the justice and legal systems. Furthermore, research has mainly focused on the bias of specific elements in the justice and legal systems, be it police, eyewitnesses, forensic evidence, jurors, or judges, but seldom a larger view of how bias cascades and snowballs through the entire justice and legal systems.

## 4. Public Policy to Minimize Bias in the Justice and Legal Systems

### 4.1. The Challenge

To minimize bias, one must first acknowledge the existence of bias and its impact and be transparent about it. The justice and legal systems cannot improve if the problems are not acknowledged. This is a huge obstacle in addressing bias, especially within the justice and legal systems because of the following:

1. The *bias blind spot* (Neal & Brodsky, 2016; Pronin et al., 2002, 2004) and the implicit nature of cognitive bias make it hard for people to acknowledge its existence, let alone to be transparent about it (which is the next best thing: if you cannot remove the source of the bias and its possible impact, at least be transparent about it).
2. Not only is the bias implicit but, specifically in the justice and legal systems, errors are not apparent. In contrast to other domains, where aircraft crash, patients die, or stocks lose value, in the justice and legal systems, the ground truth is not known, and we have no idea how many innocent people are wrongfully convicted. If/when this happens, it is not as apparent as it is in other domains.
3. The adversarial legal system makes it hard, almost impossible, to uncover and acknowledge the biases. There is a (justifiable) fear that any acknowledgment will be used against them in court. Furthermore, to avoid court exposure of existing biases in the justice and legal systems, attractive plea bargains (and even dropping all charges) are offered when the prosecution realizes that the defense is going to publicly reveal the bias against their client, especially when the bias is widespread and systemic to the entire justice and legal systems. The fear of having bias exposed (as well as errors and other issues) also makes forensic science crime laboratories reluctant to do research, validation studies, and proper quality assurance measurement (and sometimes, they stop them in the middle when they find problematic data showing biases and other issues).

These are major obstacles and challenges in confronting bias in the justice and legal systems because dealing with bias requires to first acknowledge its existence and understanding the nature of cognitive bias. I say and emphasize ‘understand the nature of cognitive bias’ because the bias fallacies (discussed earlier) prevent dealing with bias as many people incorrectly believe they can overcome bias by mere willpower or that experience and technology make them immune to bias. Only after acknowledging and understanding the problem can we then really start to address it and take practical and proper countermeasures to minimize the causes of bias at its source, or, at the very least, to minimize the bias cascade and bias snowball contaminating other elements in the justice and legal systems. Minimizing the impact of cognitive bias at its source entails avoiding (or at least minimizing) the things that trigger the bias, and this is often achieved through ‘context management’ (e.g., I. E. Dror et al., 2015; Gilbert, 1993).

### 4.2. Blinding to Irrelevant Information

If we go back to consider the sources of bias, it is clear that people in the justice and legal systems are often exposed to task-irrelevant biasing contextual information. Sometimes, this is hard to avoid, as the information triggering the bias is integrated into the actual evidence (see the sources of bias, Level 1 in Figure 1). However, often, it is possible (and even easy) to separate and manage the biasing information. Level 3 (in Figure 1) is about irrelevant contextual information, such as a fingerprint examiner knowing whether the suspect has a criminal record (this information appears in over 40% of the formal request for forensic examination, Gardner et al., 2019).

Similarly, the prosecutor at the DA Office considering whether to prosecute a case knows the race of the suspect (again, I refer the reader to the sources of bias, Level 3 in Figure 1). These types of irrelevant information are often known, and data show, for example, that the race of a suspect can bias the prosecutors’ decisions and that bias will be reduced if prosecutors do not know the race of the defendant when deciding whether or not to prosecute (Sah et al., 2015). Thus, a simple takeaway message as to how to minimize bias is: as much as possible, blind exposure to irrelevant biasing contextual information.

I say ‘as much as possible’, as sometimes, such information is in the evidence, as noted above and Level 1 of the sources of bias (Figure 1). However, even in those circumstances, one can still sometimes take measures to minimize the bias. For example, an autopsy entails exposure to the race of the deceased, which is unavoidable. However, even in such cases, a pathologist can be asked blindly (without knowing the race) to examine X-rays or CT scans, so as to make an assessment about whether injuries are consistent with a fall. Similarly, medical history can sometimes be examined blind to race (or/and other irrelevant information) to determine potential patterns of abuse.

This also enables to minimize another source of bias, the base rate bias (see Level 4 in Figure 1), as the pathologist’s past experience base rate that dead children that are black or/and brought to the hospital by the mother’s boyfriend may have historically been determined to have been more likely to have died as a result of homicide rather than an accident (I. E. Dror et al., 2021).

In the wider context of the justice and legal systems, examining the credibility of a witness can entail reading statement transcripts, rather than seeing a video that exposes the race of the person (as well as other biasing information, such as their attractiveness). In the context of the courtroom, the presence of a dog accompanying vulnerable witnesses when testifying can bias jurors’ perceptions and evaluation of the testimony and hence steps must be taken to minimize such biases (Ensminger et al., 2020). For example, rather than not allowing vulnerable witnesses to have an accompanying dog, measures can ensure that the dog’s presence is not within the jurors’ view.

### 4.3. Compartmentalization

Nevertheless, sometimes, it is not possible to blind the irrelevant contextual information. In such cases, we want to at least minimize the bias cascading and snowballing, further contaminating other elements in the justice and legal systems. Sometimes, relevant information can be biasing, in such cases, one should not blind this information (especially if it is very relevant and minimally biasing—a different conclusion may be warranted if the information is only mildly relevant but extremely biasing). If biasing information cannot be removed, regardless of whether it is relevant or not, people involved in one aspect of a case should not be exposed to unrelated findings from other aspects of the case.

Many people who are involved and contribute to the investigation are not meant to integrate different lines of evidence and therefore should only receive the information they need to do their work. Indeed, the President’s Council of Advisors on Science and Technology (2016) emphasized the need to avoid exposure to potentially biasing information, and the National Commission on Forensic Science (2015) calls for forensic analysis to be based only upon task-relevant information.

Minimizing the bias cascade and bias snowball effects requires *compartmentalization*. That, for example, the police, forensic laboratory, and prosecution will not work as a team but will rather work independently with minimal coordination and avoid creating mutually supporting biases. Indeed, many forensic crime laboratories are part of the police and work closely with the prosecution.

The findings and conclusions of experts examining the evidence feed into the police investigation. Then the police investigative findings, along with the expert evidence, should be provided to the prosecution, who then decide if and who to charge with the crime. The National Academy of Sciences (2009) recognized the need for compartmentalization by recommending to increase and maximize the independence of experts examining evidence in legal proceedings.

Interactive biasing loops in bias snowball should be avoided. Recall the example of how the District Attorney prosecution office can introduce pressure and bias to ‘re-do’ and ‘re-consider’ the work and conclusions conducted by other elements of the justice and criminal systems: such ‘teamwork’ and influences should not be allowed. The different elements (slices in the Swiss cheese model, see Figure 2) create layers of balances and checks, but only if they work independently.

To illustrate the broad existence of bias cascade and bias snowball and the wide applicability of compartmentalization as an effective approach to minimize them, consider, for example, the CSI who collects fingerprint evidence at the crime scene and then analyses that evidence back at the forensic laboratory. The comparison of fingerprints at the laboratory requires very limited and specific information (such as the friction ridge detail, the surface that the fingerprint was deposited on, and the technique used to lift the fingerprint). In contrast, the CSI at the crime scene requires a much broader set of information and context (and even if it is not required, being at the crime scene exposes them to a whole range of potentially biasing irrelevant information). When the same person who was the CSI at the crime scene is the same person doing the fingerprint comparison back at the laboratory, then the biasing elements from the crime scene cascade to bias the fingerprint comparison in the laboratory. This can easily be solved by compartmentalization, whereby people in the laboratory do not do fingerprint comparisons on evidence that they themselves collected at the crime scene.

The problem of bias cascade and bias snowball is prevalent throughout the justice and legal systems, and the approaches to minimize them, as well as other biases, involve mainly context management. In this paper, I use a variety of examples to illustrate the problems and their potential solutions, and these can be further applied across the justice and legal systems. For instance, the example above for the need to compartmentalize between the collection of evidence from the crime scene and its analysis back at the laboratory is also applicable to judiciary decision making. Judges often have to decide about the admissibility of evidence. The problem is that when they decide that it is not admissible, they have had to examine it and thus have been exposed to it. The illusion of control (see bias fallacies, above) makes it clear whether they like it or not, acknowledge it or not, that this now inadmissible evidence will still nevertheless impact (at least implicitly) their subsequent decisions and ruling. As with the CSI and BPA examples and solutions, what is needed here is that a different judge will make decisions about the admissibility of the evidence (compartmentalization), so if it is determined to be inadmissible, the presiding judge in the case will not have been exposed to it.

The examination and example of judiciary decision making, along with the CSI example (and there are many other examples across the justice and legal systems, such as jailhouse informants creating bias; Jenkins et al., 2023), all show that the biasing processes and their solutions are similar across and within very different and diverse elements of the justice and legal systems, as further detailed below.

### 4.4. Bias by Relevant Information and Linear Sequential Unmasking

Until now, I have focused mainly on ways to deal with and minimize the biasing effects of irrelevant information. However, even relevant needed information can cause bias (see, Level 2 in Figure 1). To avoid such bias (suspect/target driven bias causing circular and backward reasoning driven by the Reference Materials, see details and discussion about the sources of bias, above), procedures such as linear sequential unmasking (LSU, see also LSU-E) ensure that the actual data/evidence is driving the cognitive process, rather than the suspect (I. E. Dror et al., 2015; I. E. Dror & Kukucka, 2021). Indeed, in response to the FBI’s erroneous identification of Mayfield, the “FBI Laboratory’s increased focus on a linear approach was at least in part a response to the OIG’s findings regarding the role of circular reasoning in the Mayfield error” (OIG, 2011, p. 5).

LSU procedures mandate that information is presented in an optimized sequence. First, always start with the actual data/evidence, not the suspect, or the theory of what happened, so as to minimize preconceived notions and expectations as to what the data/evidence shows. Second, sequence information so as to start with information that is less biasing, as well as most relevant and objective. For example, a police detective or prosecutor evaluating a case should first examine the more objective evidence, e.g., look at camera video recordings evidence before reading eyewitness testimonies evidence (I. E. Dror & Kukucka, 2021; for details on practical implementation of LSU-E, see Kunkler & Roy, 2023; Quigley-McBride et al., 2022; Whitehead et al., 2022).

**Figure 4:**
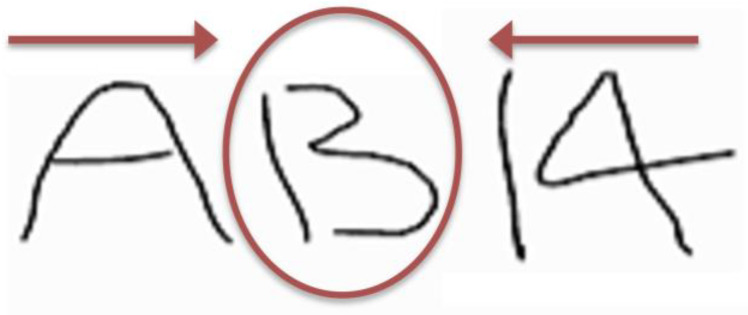
An illustration of why sequencing can be important. Reading from left to right, the first/left-most stimulus can affect the interpretation of the middle stimulus, such that it reads as A-B-14; but reading the same stimuli, from right to left, starting with 14 as the first stimulus, often makes people see the stimuli as A-13-14, i.e., the middle stimulus as a ‘13’ (or a ‘B’) depends on what you start with first (I. E. Dror & Kukucka, 2021).

The idea behind LSU and LSU-E is that the initial information has a great impact in priming and creating expectations, and thus can bias if and how subsequent information is processed (see Figure 4 for an illustration). The role and impact of sequencing have been demonstrated in a number of domains (e.g., Darley & Gross, 1983; Lund, 1925), including in the justice and legal systems, e.g., jury deliberations (Carlson & Russo, 2001; Lawson, 1967). Hence, it is important to always start with the actual data/evidence (recall the CSI example, above, whereby they are briefed about what happened before they even get to the crime scene).

The LSU-E approach is applicable throughout the justice and legal systems. Even a home visit to assess whether a child should be removed, hearing an audio recording, or watching a video (and many more examples of work conducted in the justice and legal systems) should all use LSU-E to minimize bias. LSU-E mandates always to start with the actual evidence, and that any interpretation or theory, as well as relevant contextual information, should be seen only after the actual evidence is presented and examined, and only when it is needed.

The importance and impact of sequencing have been demonstrated not only in jury decision making (Carlson & Russo, 2001; Lawson, 1967) and other domains but even in scientific evidence that is highly relied upon and used in the justice and legal systems (e.g., Davidson et al., 2023; Lynøe et al., 2017).

### 4.5. Generating a Hypothesis and Using Multiple Hypotheses

A hypothesis can create bias as it often entails confirmation and other biases that generate evidence, confirming that hypothesis while ignoring (or dismissing) conflicting evidence (Baldwin, 1993; Klayman & Ha, 1987; Wagenaar et al., 1993). Forming a hypothesis is hard to avoid; it is not only often needed but many times unavoidable. Therefore, the question is how and who generates it and based on what. Hypothesis sharing by different elements in the justice and legal systems can create a powerful bias cascade as the hypothesis is transmitted further and further. It is much better if the hypothesis at each stage is first generated by the evidence itself and then compared to the other hypotheses.

This illustrates the wide range of utility and applicability of LSU across the justice and legal systems. In this application to minimize bias cascade the hypothesis is first generated by the person examining the evidence, before exposure to the ‘reference material’, i.e., someone else’s hypothesis. For example, a CSI should first generate their own hypothesis, based on what they see at the crime scene, without initially being provided with the police hypothesis (as often happens before they even see the crime scene). This is not to prevent the police from providing the CSI with a hypothesis (just as LSU is not about preventing information, as much as it is about optimizing the order of information so as to minimize bias). Hence, the idea is to prevent the CSI from starting first with the police hypothesis but rather have them start with their own evidence-based generated hypothesis based on what they see at the crime scene. Then, the police hypothesis can be presented and considered.

To minimize bias, it is better to seriously consider other alternative hypotheses (Borum et al., 1993; O’Brien, 2009). Indeed, research shows that investigating crime with more than one hypothesis can lead to different investigative conclusions (Lidén et al., 2023). Using multiple hypotheses and conducting a *differential diagnosis* (a process of generating and considering multiple alternative hypotheses that can explain the same evidence) can reduce the bias of having a single hypothesis and erroneous conclusions (Maude, 2014).

These are only some solutions, and more have to be considered and developed. For example, how mandating attorneys and judges to have experience working for the prosecution as well as the defense can minimize the biases caused by personal factors and education (see Figure 1). It is also important to consider how to minimize one-sided and biased incentives to reach certain conclusions, such as paying forensic laboratories only for convictions (Koppl, 2020) or rewarding forensic examiners for solving crimes but not for avoiding false convictions.

Generally, minimizing biases requires multiple steps and actions:

1. Proper training and education (see Level 6 in the sources of bias, Figure 1) so the different elements in the justice and legal systems know and understand cognitive bias and acknowledge its existence and potential harm (see above, how this is a prerequisite to minimizing bias, and why it is so hard to achieve in the justice and legal systems).
2. Procedures and best practices that blind irrelevant information and manage the flow of information to avoid bias in the first place.
3. In case bias occurs, use compartmentalization and other measures to minimize it contaminating further decisions and elements in the justice and legal systems. For example, make sure that each element is organizationally separate and independent (see Level 5 in the sources of bias). Indeed, many forensic laboratories are part of the police and even part of the DA’s Office.
4. Mandating full transparency, so every and each communication between the different elements is documented. This, by itself, will make people reluctant to give irrelevant contextual information or attempt to bias others. And, if such things do occur, at least it will be transparent.

In short, the various elements in justice and legal systems need to acknowledge their biases and take measures to avoid them, but if (when) they are contaminated by bias, that these biases do not cascade and snowball. In this paper I show that different and diverse multiple decision points—within and across the various elements of the justice and legal systems—all share similar underlying biasing cognitive processes and thus similar ways to combat and minimize them.

## Data Availability

No new data were created or analyzed in this study. Data sharing is not applicable to this article.

## Note

1 This definition was originally created to address bias in forensic science; here, it is applied across the justice and legal systems.

## Public Significance Statement

The justice and legal systems suffer from explicit intentional bias but more so from widespread cognitive biases. These biases impact even objective scientific evidence, and they cascade and snowball throughout the justice and legal systems. Acknowledging these biases is a challenge that must be met before countermeasures can be put in place to minimize them.
